# An open-access plug-in program for 3D modelling distinct material properties of cortical and trabecular bone

**DOI:** 10.1186/s42490-022-00065-z

**Published:** 2022-09-24

**Authors:** Gregory R. Roytman, Matan Cutler, Kenneth Milligan, Steven M. Tommasini, Daniel H. Wiznia

**Affiliations:** 1 Yale Center for Medical Informatics, Yale School of Medicine, 300 George St, New Haven, CT 06511 USA; 2 VA Connecticut Healthcare System, Veterans Health Administration, 950 Campbell Ave, West Haven, CT 06516 USA; 3 Orthopedics and Rehabilitation, Yale School of Medicine, 47 College Place, New Haven, CT 06510 USA; 4 Biomedical Engineering, Yale School of Engineering & Applied Science, 17 Hillhouse Avenue, New Haven, CT 06520 USA; 5 IsoPlexis Proteomic Solution, 35 NE Industrial Rd, Branford, CT 06405 USA; 6 Mechanical Engineering & Materials Science, Yale School of Engineering & Applied Science, 17 Hillhouse Avenue, New Haven, CT 06520 USA

**Keywords:** Materials, Finite element analysis, Computed tomography, Orthopedics, Simulation, In-Silico, Modeling

## Abstract

**Background:**

Finite element modelling the material behavior of bone in-silico is a powerful tool to predict the best suited surgical treatment for individual patients.

**Results:**

We demonstrate the development and use of a pre-processing plug-in program with a 3D modelling image processing software suite (Synopsys Simpleware, ScanIP) to assist with identifying, isolating, and defining cortical and trabecular bone material properties from patient specific computed tomography scans. The workflow starts by calibrating grayscale values of each constituent element with a phantom – a standardized object with defined densities. Using an established power law equation, we convert the apparent density value per voxel to a Young’s Modulus. The resulting “calibrated” scan can be used for modeling and in-silico experimentation with Finite Element Analysis.

**Conclusions:**

This process allows for the creation of realistic and personalized simulations to inform a surgeon’s decision-making. We have made this plug-in program open and accessible as a supplemental file.

**Supplementary Information:**

The online version contains supplementary material available at 10.1186/s42490-022-00065-z.

## Background

The methods of modeling bone, in considering the differing material properties between trabecular to cortical bone, has been discussed and debated at length for decades in the literature [1–5]. The debate chiefly concerns the structures, densities, and Young’s Moduli of trabecular and cortical bone. Trabecular bone is primarily a spongy and anisotropic material, meant for transferring loads from articular surfaces to the denser cortical bone [2]. Cortical bone, however, is more consistent in density and stiffness, being more necessary for handling higher stressors from repeated loads of tension and compression [4, 5]. This leads authors to determine a variety of equations based on power law regressions for the determination of Young’s Modulus for the more variable spectrum of trabecular bone [1, 2], while cortical bone is usually represented with a constant Young’s Modulus [4].

The ability to define these material properties accurately in mathematical models is invaluable to translating medical device design and surgical principles to clinical applications. Orthopedic medical devices restore a patient’s function by providing an environment for bone healing or joint function. Surgeons make their best predictions for the optimal implant choice based on a patient’s bone quality, comorbidities, and their previous experiences with an implant. Accurate computational models allow surgeons and medical device engineers to simulate the performance of each implant type within a patient’s bone to make informed decisions regarding implant design, selection and surgical technique [6, 7].

We wish to present a methodology in which the previously used method of determining bone mineral density using Quantitative Computed Tomography (QCT) [8] is applied to determine material properties in Finite Element Analysis (FEA). Although modeling based on CT scans itself is not novel [1, 2, 8, 9], we incorporate this methodology into our work as a streamlined workflow with existing modeling software for convenient clinical and research applications.

**Fig. 1:**
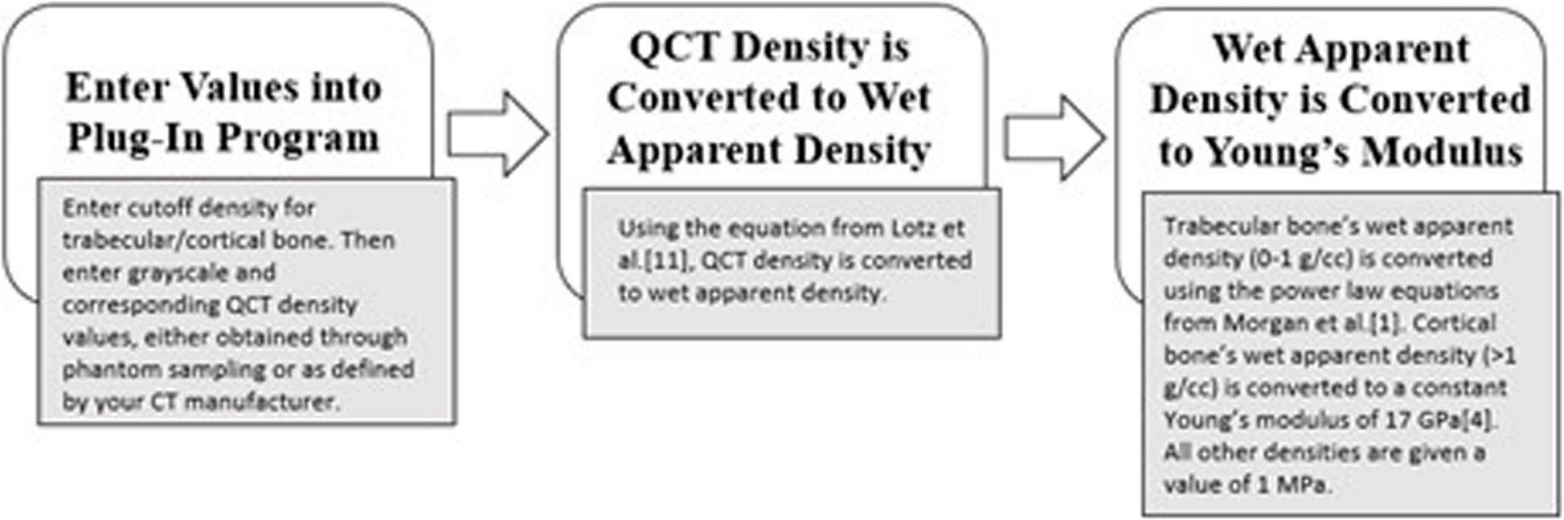
Flowchart of methodology of our plug-in program

Our workflow preprocesses Computed Tomography (CT) scans of bones using Synopsys® Simpleware ScanIP software and its Python scripting tool, produced by our lab in coordination with the Synopsys® Simpleware engineering team. As a supplemental file in this publication, we share the Plug-In Program (PIP) as open access as a supplement of this paper. Although, ScanIP is a versatile and effective tool for modeling structure as well as material properties, conversions are made from density to Young’s Modulus all using a power law equation, which we deem to be inappropriate for calculating cortical bone due to the above-mentioned differences. Although Morgan et al. [1] describe a power law equation for density to modulus conversion, they were created with trabecular bone in mind. For cortical bone, therefore, we consider a constant modulus as determined by Reilly and Burstein [4]. In the following work, we describe how grayscale values from individual CT elements, or voxels, are transformed into Young’s Moduli in a three-step process (Fig. 1). First, the user enters a cutoff density for trabecular/cortical bone as well as corresponding grayscale and QCT density values. A way of obtaining grayscale and QCT density values are outlined in the Methods Section. Second, the QCT values of the Digital Imaging and Communications in Medicine (DICOM) format are converted to a wet apparent density. Finally, the wet apparent density values are converted to a Young’s Modulus based on the corresponding tissue. The adjusted DICOM files can then be used to create a 3D model and subsequent FEA.

### Computed tomography

Computed tomography (CT) scans are composed of x-rays from various angles, which are then transformed into cross-sectional images through computer processing. Series of two-dimensional images made of pixels are used to represent three dimensional volumes, commonly known as voxels, of the scanned subject. These series are typically stored as Digital Imaging and Communications in Medicine (DICOM) files, a common means for storing and transmitting medical imaging data such as CT scans. The values of the radiodensities are measured in Hounsfield units (HU). HU quantify the linear attenuation; the number of x-rays emitted by a CT scanner that are absorbed or scattered per unit thickness of the sample. HU are based on reference values for the linear attenuation at standard temperature and pressure of water (0 HU), and air (− 1000 HU) [10].

$$HU=1000\ \left(\frac{\mu -{\mu}_{water}}{\mu_{water}-{\mu}_{air}}\right)$$

While HU are negative if the radiodensity of the sample is less than that of water, radiodensities in CT images are stored only as positive values. Observed HU are first scaled using a rescale intercept (b) and rescale slope (m) before they are stored in the DICOM.

$$HU=m\ast stored\ value+b$$

The DICOM metadata stores the rescale intercept and slope under tags (0028,1052) and (0028,1053) (“DICOM Tags”). The values we manipulate in our PIP are the DICOM values (adjusted HU), and not the HU. Due to how the values are transformed to CT densities, the original unit does not influence the relationships as long as internal consistency is maintained.

### Types of densities that are obtained from CT

Our workflow requires conversion to a density in each voxel based on the grayscale image that most closely reflects what its real-world density might be. In considering that the density of the physical bone cannot be measured to complete accuracy, we term various densities as calculated densities to describe what we would obtain for individual voxels of data. Wet apparent density describes the wet mass divided by the bulk volume of a sample in a voxel, from which we can obtain Young’s Moduli values [8]. This is calculated through a power law regression, as described by Morgan et al. [4]. Lotz et al. [11] describes a method of obtaining wet apparent density values through a linear regression equation with quantitative CT (QCT) density. QCT values describe the bone mineral density of bone structure in each voxel of an image [8]. The benefits of QCT density allow a detailed map of bone density, to the extent that trabecular bone can be distinguished from cortical bone [8].

## Implementation

**Fig. 2:**
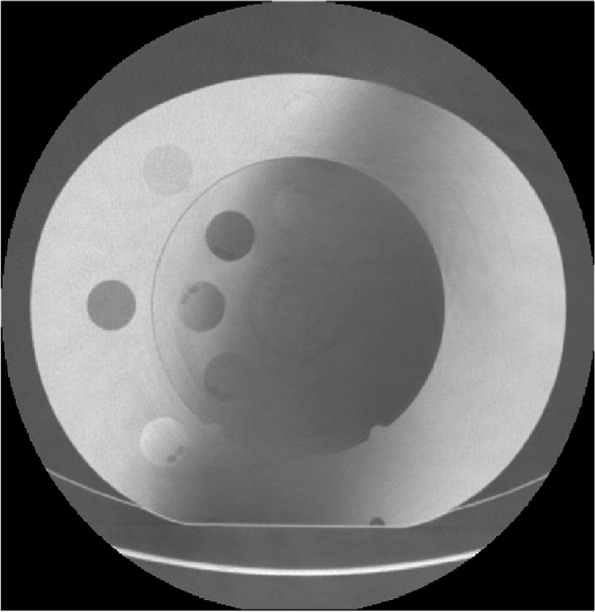
A DICOM image with a large artifact from the study’s phantom

The DICOM from scanning the phantom (Fig. 2) was imported to Synopsys® Simpleware ScanIP software, where the phantom was segmented into cylinders/disks containing each sample. ScanIP’s grayscale measurement feature was then used to measure the average grayscale of each sample. The phantom should, ideally, be scanned along with the anatomy in question to reduce any potential variables and improve accuracy. ScanIP’s grayscale feature measures in HU and the results are converted to the DICOM’s stored grayscale values. By sampling the HU of the volume of an entire phantom (these need to be defined as tissue equivalent electron density samples), the impact of noise is reduced, and the results may more accurately represent the measured radiodensities for the entire phantom.

The sampling of each grayscale value in the phantom was performed using ScanIP’s Profile Line Measurement tool. This tool allows one to draw a line through a sample, export its grayscale values, and calculate an average for that sample’s grayscale. Any user of ScanIP can use the Profile Line tool, find grayscale values manually by hovering, or calculate them using several other available measurement methods (including the ability to calculate average grayscale values over a volume). After the user starts the PIP, it then prompts the user to enter these grayscale values and each phantom’s corresponding manufacturer defined QCT density. This will be specific to each individual PIP user’s CT scanner and phantom.

**Fig. 3:**
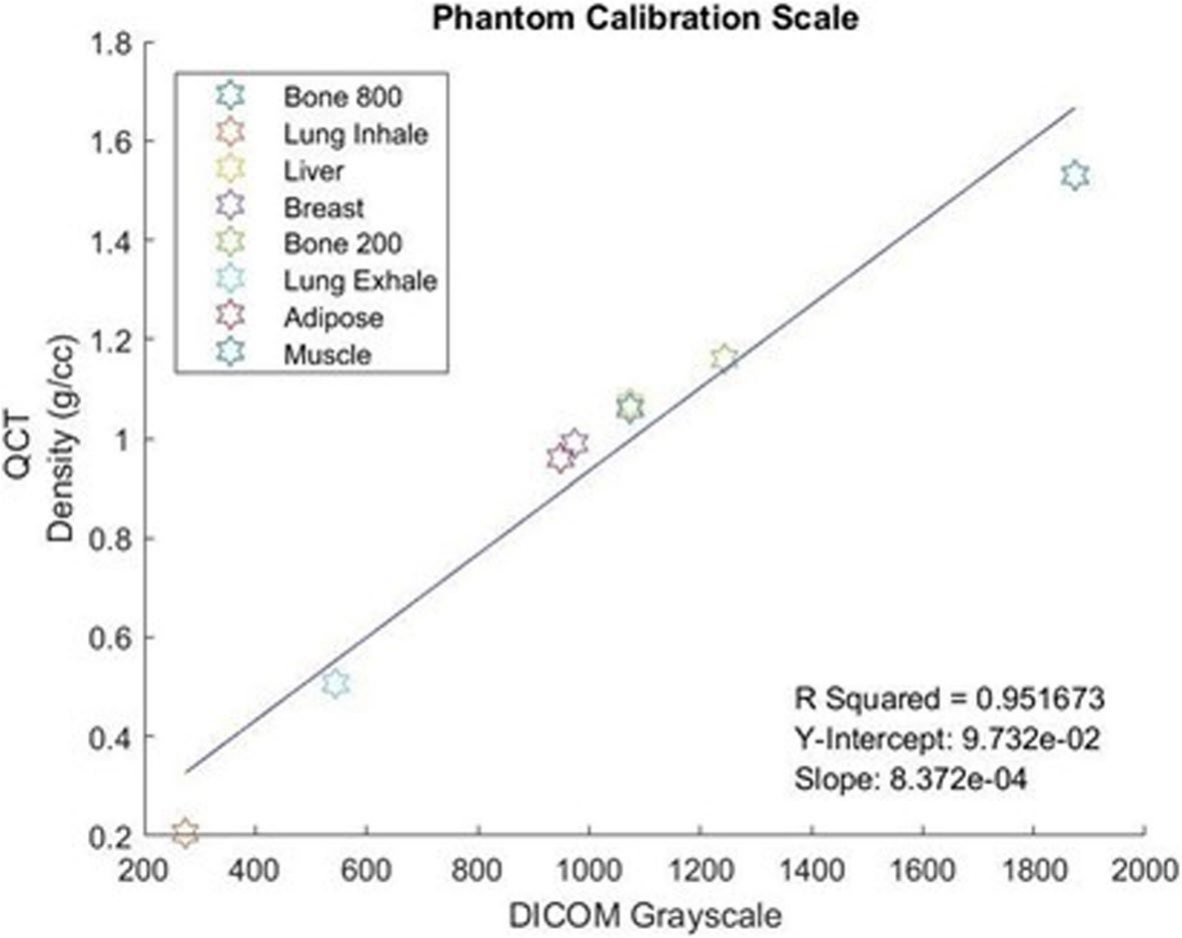
DICOM value scale based typical grayscale values

A linear regression was then performed by the PIP using the QCT density and the DICOM grayscale values of the phantom. This gives us an equation which relates the DICOM grayscale values to the QCT density. A sample plot of values and the y-intercept and slope can be seen in Fig. 3. Each voxel within our DICOM was then converted from grayscale to QCT density using this linear regression equation. The QCT density values were then converted to wet apparent density based on the equation below, established by Lotz et al. [11].

$${\rho}_{apparent}\ \left(\frac{g}{cm^3}\right)=0.0012\ {\rho}_{CT}+0.17$$

The wet apparent density was then converted to Young’s Modulus by the PIP based on three definitions that are applied to the dataset:

For air pockets, where ρapp< 0 g/cc:

$$E=1\ MPa$$

For trabecular bone, where 0 < ρapp < 1 g/cc:

$$E=\mathrm{11,417.6}\ {\rho}^{1.89}\ MPa$$

For cortical bone, where ρ_app_ > 1 g/cc:

$$E=\mathrm{17,000}\ MPa$$

The first equation ensured that air pockets maintain a realistically low Young’s Modulus. The second equation is the power-law equation for trabecular bone [1] (adjusted by a factor of 1.28 to account for transverse stiffness differentce in trabecular bone [12]) and the third is the uniform Young’s Modulus for cortical bone as described by Reilly and Burstein [4]. Although we choose 1 g/cc as our cut-off density due to its close approximation to the difference in densities of trabecular and cortical bone [12], we have given users the option to set their own cortical cut-off density. Each voxel was then converted to Young’s Modulus accordingly in the PIP, based on its wet apparent density.

## Results

**Fig. 4:**
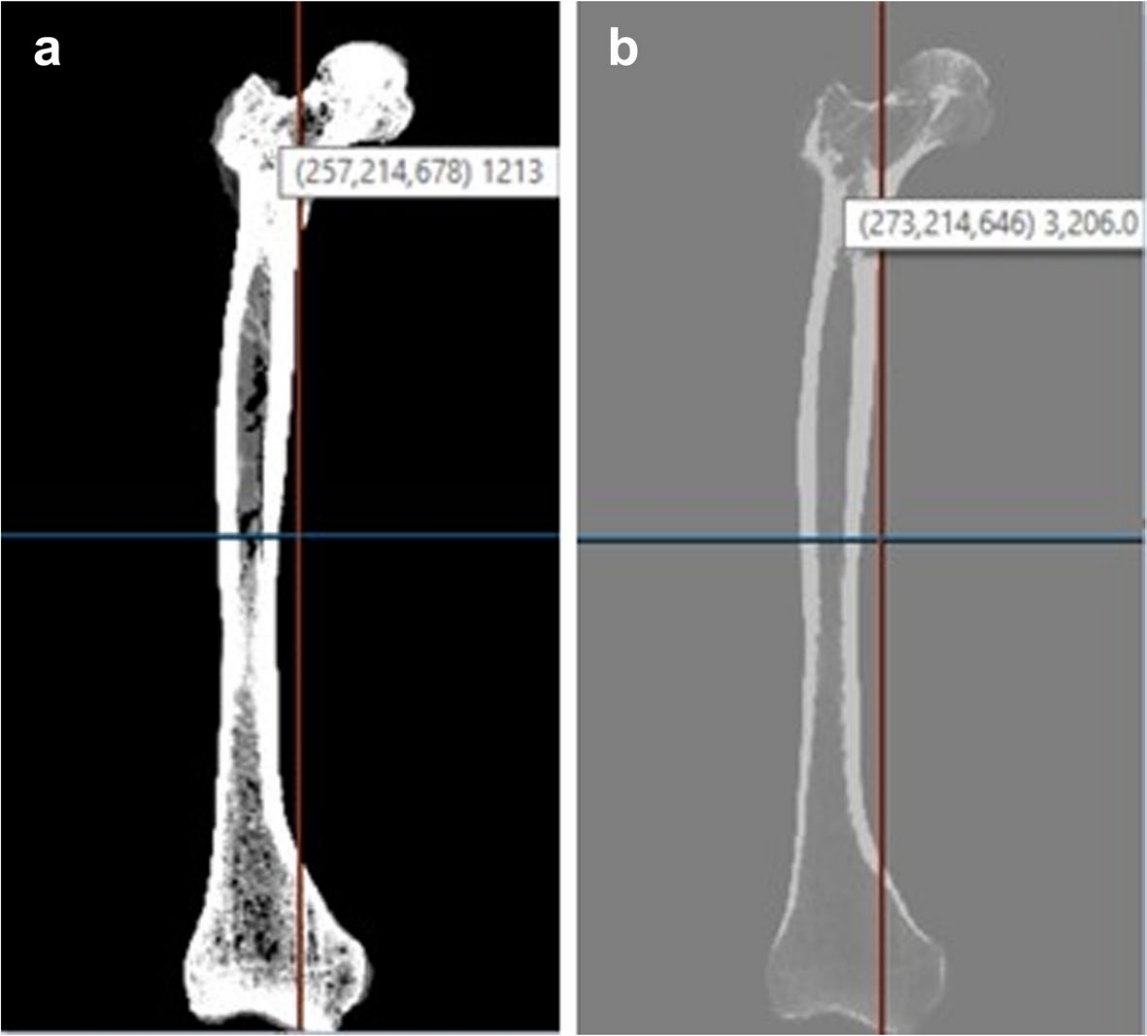
(**a**) Left. Original CT scan prior to processing using our PIP in the original grayscale (x,y,z, grayscale) (**b**) Right. CT scan after processing using our PIP in Young’s Modulus (MPa) (x,y,z, Young’s Modulus)

We used CT scans of a phantom obtained from CIRS Tissue Simulation and Phantom Technology, scanned by a LightSpeed VCT scanner manufactured by GE Medical Systems. The femur was obtained from a 70-year-old 5 ft. and 6 in tall patient. Figure 4a shows the patient’s original CT scan in its original grayscale and Fig. 4b shows the CT scan after processing using our PIP.

## Discussion

### Research implications and next steps

The resulting calibrated CT scan, containing the Young’s Moduli of each of the voxels, will allow the use of patient’s imaging data more flexibly for personalized biomechanical FEA simulation of surgical implants. This will create a more accurate model of cortical bone than using the extant power law conversion from density to Young’s Modulus that currently exists. Although appropriate for trabecular bone [1], the power law equation is inappropriate for the constant modulus of cortical bone [4]. The results of cortical bone Young’s Modulus analysis by Hamed et al. bears out the near constancy and transversely isotropic nature of cortical bone, despite some local variation [13], which approximates the constant modulus we set in our PIP. This also provides the user a more convenient method of modeling than ScanIP’s masking tool does, alone, automating the process of filtering cortical and trabecular bone by density.

Previous studies within the discipline of orthopedics have demonstrated the benefit and improved outcomes that come with personalized in silico simulations [6, 7, 14, 15]. Our analysis of the femur in Fig. 4 provides the user, for either research or clinical purposes, a one-to-one voxel-to-voxel view between the original density of the scan and resulting Young’s Modulus. In silico biomechanical tests can therefore be performed prior to surgery, for which knowing Young’s Moduli are crucial.

Previous studies have examined the material properties of femurs sampled from different sites from the femoral neck and greater trochanter [1, 2]. Each of them acknowledges the need to determine moduli of cortical and trabecular bone separately because of their different properties in these anatomical locations. The personalized approach and program which we describe here is superior in its pre-processing of the CT scan to produce values of Young’s Modulus for each voxel. Individual users of the PIP can enter data pertinent to their phantom and their CT scanners, providing a truly personalized application. Once moduli are obtained, our group can easily add proposed implants to a 3D rendering of the patient’s anatomy and the scan can be processed by finite element software. This resulting simulation will provide invaluable information for surgeons to use in planning, invariably leading to improved outcomes.

### Limitations

When examining values of CT scans two factors must be considered: scanner variation and noise. Noise describes the random variances in measured HU across a uniform sample. Noise can cause two voxels of the same tissue to be measured as having different HU [16]. For Radiologists, noise can be a significant issue when examining samples with fine elements, such as blood vessels. In bone, the obscuring of fine details may not be as significant a concern. However, noise can have an impact on the DICOM-QCT density scale creation process.

In addition, variations between scanners can impact the HU measured. Different models of scanners often record different HU values for the same sample. According to one study, the radiodensity of a sample measured across two scanners differed by almost 50 HU, twice the difference between adipose and breast tissue or half the difference between muscle and liver tissue [17]. Scanner variations can be accounted for by calibrating the particular scanner you are using to a phantom as well as scanning samples and phantoms together.

While developing this method, a use case scenario was only performed using a single cadaveric femur. Although it shows that the PIP can work on scans, more rigorous and wider implementation of the PIP is warranted. This limitation requires that this method continues to be used, validating it using live patient CT scans and applying it in finite element analysis. We recommend such application in future studies using the PIP.

## Conclusions

The Plug-In file described here can effectively process and convert grayscale data to Young’s Modulus data per voxel to provide for convenient assignment of material properties in 3D modeling of CT scanned bony anatomy. This will allow for accurate in-silico simulation and give surgeons a more accurate understanding of a patient’s anatomy prior to surgery.

### Availability and requirements

Project Name: Yale Material Properties ScanIP Plug-In.

Project home page: https://sourceforge.net/projects/yale-scanip-plug-in/files/

Operating system(s): Platform independent.

Programming language: Python.

Other requirements: Synopsys® Simpleware ScanIP Software.

License: License for ScanIP software required.

Any restrictions to use by non-academics: License listed above is needed.

## Supplementary Information

**Additional file 1:** **Supplementary file**. Source Code. Python code used for the Plug-In tool for giving the resulting Young’s Modulus for a CT scan.

## Data Availability

An up-to-date version of our Plug-In can be found at: https://sourceforge.net/projects/yale-scanip-plug-in/files/.

## Abbreviations

CT: Computed Tomography
DICOM: Digital Imaging and Communications in Medicine
FEA: Finite Element Analysis
HU: Hounsfield Units
PIP: Plug-In Program
QCT: Quantitative Computed Tomography
