# Supplementary material for: An open-access plug-in program for 3D modelling distinct material properties of cortical and trabecular bone
Source: BMC Biomed Eng. 2022 Sep 24;4:8. doi: 10.1186/s42490-022-00065-z (PMC9509591; doi:10.1186/s42490-022-00065-z)
Supplement: Supplementary file 1 — Additional file 1: Supplementary file. Source Code. Python code used for the Plug-In tool for giving the resulting Young’s Modulus for a CT scan. [file 42490_2022_65_MOESM1_ESM.docx]

**Supplementary File: Source Code**

An up-to-date version of this code can be found at: <https://sourceforge.net/projects/yale-scanip-plug-in/files/>

from scanip_api3 import *

import os

# Example Command

# This has been modified from the Command and UserAction example in the Help guide

# This script pops up an information dialog

# Linear Regression

def LinReg(sampleGSAvgs, sampleQCTs, numSamples):

GS_mult_QCT = [] # element-wise multiplication

GSsq = []

QCTsq = []

for i in range(1, numSamples):

GS_mult_QCT.append(sampleGSAvgs[i] * sampleQCTs[i])

GSsq.append(sampleGSAvgs[i] ** 2)

QCTsq.append(sampleQCTs[i] ** 2)

yInt = ((sum(sampleQCTs) * sum(GSsq)) - (sum(sampleGSAvgs) * sum(GS_mult_QCT))) / ((numSamples * sum(GSsq)) - (sum(sampleGSAvgs) ** 2))

slope = ((numSamples * sum(GS_mult_QCT)) - (sum(sampleGSAvgs) * sum(sampleQCTs))) / ((numSamples * sum(GSsq)) - (sum(sampleGSAvgs) ** 2))

return yInt, slope

class CalibrationCommand(Command):

# Initialisation for the ThresholdCommand, saves a copy of the specified mask

# for use in the Undo method.

def __init__(self):

Command.__init__(self)

self.doc = App.GetInstance().GetActiveDocument()

self.background = App.GetInstance().GetActiveDocument().GetActiveBackground()

# The name of the command, this appears in the Undo menu item's description.

def GetName(self):

return "Command Example"

# Performs the actual operation.

def Do(self):

# Duplicate the currently active background, creating a 32bit floating point duplicate for processing

new_background = self.background.DuplicateAs(Doc.Float32,

self.background.GetPixelRange(self.background.GetPixelType()),

self.background.GetPixelRange(self.background.GetPixelType()))

#Set the name to something meaningful

new_background.SetName("Calibrated DICOM")

backgroundPosition = new_background.GetVoxels()

# Obtain Cortical/Trabecular Density Cutoff From User

cutoffDensity = InputDialog_GetDecimal("Trabecular/Cortical Cutoff", "Density (g/cc)")

# Obtain Tissue Samples

numSamples = InputDialog_GetInteger("Number of Tissue Samples", "Number of Samples:")

sampleGSAvgs = []

sampleQCTs = []

for i in range(1, numSamples+1):

sampleGSAvgs.append(InputDialog_GetDecimal("Grayscale Average of Sample "+str(i), "Grayscale Average"))

sampleQCTs.append(InputDialog_GetDecimal("QCT Value of Sample "+str(i), "QCT Value"))

yInt, slope = LinReg(sampleGSAvgs, sampleQCTs, numSamples)

while not backgroundPosition.IsPastLastVoxel():

# Get the current GS value

img_GS = backgroundPosition.GetCurrentReal()

# Convert GS to QCT to Wet Apparent

img_QCT = (yInt + (img_GS * slope) ) * 1000

img_wetapparent = (0.0012*img_QCT) + 0.17

# Sort Elastic Moduli based on Wet Apparent Density

if img_wetapparent < 0:

img_MPa = 1

elif (img_wetapparent > 0) and (img_wetapparent < cutoffDensity):

img_MPa = round((11417.6 * (img_wetapparent ** 1.89))) # Morgan 2003

elif img_wetapparent > cutoffDensity:

img_MPa = 17000 # Reilly and Burstein 1975

else:

img_MPa = 0

# set the new value

backgroundPosition.SetCurrentValue(img_MPa)

# iterate the voxel iterator class

backgroundPosition.MoveForward()

return True

# This method must be present when implementing custom commands, it allows ScanIP

# to cleanup the memory associated with your custom command once it is no longer

# required.

def OnNativeDelete(self):

self.doc.ReleaseCommand(self)

class CalibrateAction(UserAction):

""" A user action for a pop up information """

def __init__(self):

UserAction.__init__(self, "Image processing", "Additional", "Yale Material Processing")

tool_icon = os.path.join(

App.GetInstance().GetInstallLocation(), "Plugins", "Yale Logo.png"

)

if os.path.isfile(tool_icon):

self.SetBitmapFileName(tool_icon)

def OnActivated(self):

App.GetDocument().SubmitCommand(CalibrationCommand())

def main():

#App.GetInstance().GetActiveDocument().SubmitCommand(CommandExample())

App.GetInstance().AddUserAction(CalibrateAction())

main()
